# Fecal Pharmacokinetics and Gut Microbiome Effects of Oral Omadacycline Versus Vancomycin in Healthy Volunteers

**DOI:** 10.1093/infdis/jiad537

**Published:** 2023-12-05

**Authors:** Jinhee Jo, Chenlin Hu, Khurshida Begum, Weiqun Wang, Thanh M Le, Samantha Agyapong, Blake M Hanson, Hossaena Ayele, Chris Lancaster, M Jahangir Alam, Anne J Gonzales-Luna, Kevin W Garey

**Affiliations:** Department of Pharmacy Practice and Translational Research College of Pharmacy, University of Houston; Department of Pharmacy Practice and Translational Research College of Pharmacy, University of Houston; Department of Pharmacy Practice and Translational Research College of Pharmacy, University of Houston; Department of Pharmacy Practice and Translational Research College of Pharmacy, University of Houston; Department of Pharmacy Practice and Translational Research College of Pharmacy, University of Houston; Department of Pharmacy Practice and Translational Research College of Pharmacy, University of Houston; UTHealth Houston School of Public Health, University of Texas Health Science Center at Houston, Houston, Texas; UTHealth Houston School of Public Health, University of Texas Health Science Center at Houston, Houston, Texas; Department of Pharmacy Practice and Translational Research College of Pharmacy, University of Houston; Department of Pharmacy Practice and Translational Research College of Pharmacy, University of Houston; Department of Pharmacy Practice and Translational Research College of Pharmacy, University of Houston; Department of Pharmacy Practice and Translational Research College of Pharmacy, University of Houston

**Keywords:** microbiome, *Clostridioides difficile* infection, metagenomics, human, clinical trial

## Abstract

**Background:**

*Clostridioides difficile* infection (CDI) is a common healthcare-associated infection with limited treatment options. Omadacycline, an aminomethylcycline tetracycline, has potent in vitro activity against *C difficile* and a low propensity to cause CDI in clinical trials. We aimed to assess fecal pharmacokinetics and gut microbiome effects of oral omadacycline compared to oral vancomycin in healthy adults.

**Methods:**

This was a phase 1, nonblinded, randomized clinical trial conducted in healthy volunteers aged 18–40 years. Subjects received a 10-day course of omadacycline or vancomycin. Stool samples were collected at baseline, daily during therapy, and at follow-up visits. Omadacycline and vancomycin stool concentrations were assessed, and microbiome changes were compared.

**Results:**

Sixteen healthy volunteers with a mean age of 26 (standard deviation [SD], 5) years were enrolled; 62.5% were male, and participants’ mean body mass index was 23.5 (SD, 4.0) kg/m^2^. Omadacycline was well tolerated with no safety signal differences between the 2 antibiotics. A rapid initial increase in fecal concentrations of omadacycline was observed compared to vancomycin, with maximum concentrations achieved within 48 hours. A significant difference in alpha diversity was observed following therapy in both the omadacycline and vancomycin groups (*P* < .05). Bacterial abundance and beta diversity analysis showed differing microbiome changes in subjects who received omadacycline versus vancomycin.

**Conclusions:**

Subjects given omadacycline had high fecal concentrations with a distinct microbiome profile compared to vancomycin.

**Clinical Trials Registration:**

NCT06030219.

## Teaser

Omadacycline, an aminomethylcycline tetracycline, was demonstrated to have a distinct and protective gut microbiome profile and similarly achieve high fecal concentrations compared to vancomycin.

*Clostridioides difficile* infection (CDI) is a common healthcare-associated infection in the United States (US) causing nearly 500 000 infections annually [1]. CDI pathophysiology includes a significant disruption of gut microbiota, usually by broad-spectrum antibiotics, leading to loss of colonization resistance to *C difficile* [2–5]. Preferred CDI antibiotic treatments are limited to 2 oral agents: vancomycin or fidaxomicin [6]. Vancomycin is the most commonly used, guideline-preferred antibiotic but is associated with an unacceptably high recurrence rate due to its broad spectrum of activity on the gut microbiota [7, 8]. New drug development for CDI-directed antibiotics is needed. Ideal drug candidates for CDI should have potent activity against *C difficile*, high colonic concentrations when administered via oral or intravenous (IV) routes, and a lower degree of gut microbiota disruption compared to vancomycin.

Omadacycline is a novel aminomethylcycline tetracycline available in both IV and oral formulations and is approved by the US Food and Drug Administration for community-acquired bacterial pneumonia and acute bacterial skin and skin structure infections in adults. Previous studies have demonstrated its potent in vitro activity against *C difficile* including the hypervirulent ribotype 027 strain and strains resistant to other CDI-directed antibiotics [9, 10]. Hamster models of CDI have also shown favorable outcomes with omadacycline compared to vancomycin including less disruption to the gut microbiota than vancomycin [11, 12]. In clinical trials, no CDI cases were reported in patients receiving omadacycline in phase 3 studies [13]. However, fecal pharmacokinetics (PK) of oral omadacycline have not been fully determined and microbiome changes in humans have not been measured. The purpose of this phase 1, healthy volunteer clinical trial was to determine fecal PK characteristics and gut microbiome changes with omadacycline compared to vancomycin.

## METHODS

### Study Design

This phase 1, randomized study was conducted at the University of Houston College of Pharmacy, with subjects recruited between 2020 and 2021. The study was conducted in compliance with International Council for Harmonization Good Clinical Practice guidance and the ethical principles of the Declaration of Helsinki and was approved by University of Houston Committee for the Protection of Research Subjects (IRB00002009). Written informed consent was collected from each subject during the screening visit. Subjects were given unique study identifiers to ensure their privacy.

### Study Drugs

Omadacycline 150 mg tablets (lot W003167) were provided by Paratek Pharmaceuticals Inc (Boston, Massachusetts), and commercially available vancomycin hydrochloride 125 mg capsules (lot V021008) were purchased by the study investigators (PAI Pharmaceuticals, Greenville, South Carolina). All study drugs were stored in a locked cabinet at the study site and a dispensing log was maintained during the study period.

### Study Population

Eligible subjects included adults aged 18–40 years without a history of cardiovascular disease, gastrointestinal disease, hepatic disease, renal disease, diabetes, depression, vertigo, tinnitus, or diminished hearing, and who were not current tobacco users. Participants were excluded if they received an antibiotic within 90 days prior to enrollment, were currently taking anticoagulants or probiotics, or were pregnant. Subjects with allergies to study medications were excluded.

Enrolled subjects were assigned using a random number generator to receive a 10-day course of either omadacycline given once daily or vancomycin given 4 times daily. Omadacycline 450 mg (3 tablets) were given on days 1 and 2 followed by 300 mg (2 tablets) for the remaining 8 days while the subjects in vancomycin group received 125 mg (1 capsule) 4 times daily for 10 days. Omadacycline and the first daily dose of vancomycin was administered using directly observed therapy at each visit. Subjects were asked each day if they missed any of the remaining doses of vancomycin and answers were recorded in a study log.

### Safety Assessment

Safety endpoints included the rate of adverse events (AEs), which were assessed at each visit from the time of enrollment. All AEs were assessed by investigators in terms of duration, severity (mild, moderate, severe), and possible relation to study medications (not suspected, not related, unlikely related, suspected, possibly related, or definitely related).

### Sample Collection

Stool samples were collected at baseline prior to dosing (day 0), daily during therapy (days 1–10), and at 2 follow-up visits (days 13–14 and days 30–32). All samples were processed immediately following collection at the central laboratory located in the University of Houston College of Pharmacy and stored at −80°C until analyses.

### Stool DNA Extraction

Stool DNA extraction was performed using the MagAttract Power Microbiome Kit (Qiagen, catalog number 27500-4-EP) per the manufacturer’s instructions. Eluted DNA was stored at −80°C for downstream analyses.

### Quantitative Polymerase Chain Reaction Analysis

Extracted stool DNA was assayed using the Qubit 4 Fluorometer (Thermo Fisher Scientific, Waltham, Massachusetts) for quantity and quality. Stool DNA was diluted with polymerase chain reaction (PCR)–grade water to 5 ng/µL. The DNA levels of bacterial groups were assessed using specific PCR primers/conditions as previously described [14–17]. Quantitative PCR (qPCR) was performed using the QuantStudio 5 (Applied Biosystems, Waltham, Massachusetts). Each sample was prepared in triplicate with a final volume of 20 µL containing 25 ng DNA template, primers at 0.5 µM, and QuantiTect SYBR Green Mixes (Qiagen, Hilden, Germany). For Eubacteria, an FAM-tagged probe at 0.25 µM and TaqPath ProAmp Master Mixes (Qiagen, Hilden, Germany) were used. Threshold cycle values in copies per nanogram of DNA were determined using a standard curve. Standards were prepared by performing PCR using species-specific primers on corresponding bacterial strains of stool DNA. A range of 10-fold serially diluted plasmid standard DNA copies (5 × 10^8^ to 500 copies) was run on each qPCR plate in triplicate. Standard curve *R*^2^ values and copies per gram of stool were calculated using the initial DNA concentrations and stool aliquot weights.

### Metagenomic Analysis

The V4 region of the 16S ribosomal RNA gene was amplified using the dual indexing sequencing strategy as previously described [18]. Sequencing was performed using the Illumina MiSeq (Illumina, San Diego, California) with a MiSeq Reagent Kit V2 for 500 cycles. Sequences yielding >5000 reads per sample were used for operational taxonomic unit clustering using the CLC Genomics Workbench v22.0.2 (Qiagen, Hilden, Germany).

### Fecal PK Analysis

Fecal samples from the vancomycin group were treated with 1 mL of extraction solvent as previously described [19]. The sample mixture was vortexed for 10 second, ultrasonicated for 5 minutes, and centrifuged at 10 000*g* for 3 minutes. The supernatant was obtained and fecal vancomycin concentrations were quantified using the Shimadzu Nexera-i LC-2040C 3D Plus high-performance liquid chromatography system with a limit of quantification (LOQ) of 0.4 µg/mL. Fecal omadacycline concentrations were quantified using a validated liquid chromatography–tandem mass spectrometry method. The LOQ was 0.1 ng/mL.

### Statistical Analysis

Safety, tolerability, and PK data were summarized and tabulated using descriptive statistics as appropriate. Quantitative changes in bacterial taxa concentrations (qPCR) or relative abundance (metagenomics) were summarized as mean with standard deviation (SD) or median with interquartile range as appropriate and compared using the Wilcoxon rank-sum test between groups or repeated-measures analysis. All analyses were conducted using SAS version 9.4 (SAS Institute, Cary, North Carolina) or R (v4.2.2; R Core Team 2021) software [20]. Microbiome data were analyzed using vegan R package (v2.6.2; Oksanen 2022) and visualized using ggplot2 R package (v3.3.6; H. Wickham 2016). Statistical tests were performed via rstatix R package (v0.7.0; Kassambara 2021). A *P* value <.05 was considered significant.

## RESULTS

Sixteen healthy subjects, with a mean age of 26 (SD, 5) years and an mean body mass index of 23.5 (SD, 3.9) kg/m^2^ were enrolled (Table 1). Most subjects were male (62.5%) and either White (37.5%) or Asian (37.5%). The majority of subjects (87.5%) were omnivores, and none used tobacco products. All enrolled subjects completed the study, including the follow-up. Omadacycline was well tolerated with a similar AE profile compared to vancomycin (Table 2). A total of 18 AEs were reported from 12 subjects, with 7 in the omadacycline group and 5 in the vancomycin group. The severity of all AEs was either mild (n = 16) or moderate (n = 2) and their duration did not exceed 24 hours. Of the 15 AEs categorized as suspected or possibly related to the study drug, 13 (86.7%) were nausea or vomiting (10 reports from the omadacycline group and 3 reports from the vancomycin group). All AEs occurred equally in both groups, except vomiting, which only occurred in the omadacycline group. All AEs resolved without therapeutic interventions and did not require discontinuation of therapy.

**Table 1.** Baseline Characteristics of Subjects by Antibiotic Group

Characteristic	Omadacycline (n = 8)	Vancomycin (n = 8)
Age, y, mean ± SD	27.5 ± 4.2	24.6 ± 5.6
Sex, No. (%)		
Male	5 (62.5)	5 (62.5)
Female	3 (37.5)	3 (37.5)
Race, No. (%)		
White	2 (25.0)	4 (50.0)
Asian	3 (37.5)	1 (12.5)
African American	3 (37.5)	3 (37.5)
Weight, kg, mean ± SD	68.2 ± 15.1	72.7 ± 19.4
BMI, kg/m^2^, mean ± SD	23.1 ± 3.8	23.9 ± 4.2
Dietary habits, No. (%)		
Omnivore	7 (87.5)	7 (87.5)
Vegetarian	1 (12.5)	1 (12.5)

Abbreviations: BMI, body mass index; SD, standard deviation.

**Table 2.** Summary of Adverse Events in Subjects Given Omadacycline or Vancomycin

Adverse Event	Omadacycline (n = 8)	Vancomycin (n = 8)
No. of subjects with ≥1 AE, No. (%)	7 (87.5)	5 (62.5)
Mild AE (grade 1)	5 (71.4)	5 (100.0)
Moderate AE (grade 2)	2 (28.6)	0 (0)
Severe AE (grade 3)	0 (0)	0 (0)
Serious AE	0 (0)	0 (0)
No. of drug-related AEs^a^, No. (%)	11	4
Nausea	6 (54.5)	3 (75.0)
Vomiting	4 (36.4)	0
Dyspepsia	1 (9.1)	1 (25.0)

Abbreviation: AE, adverse event.

^a^Categorized as suspected or possibly related AE (n = 15).

### Fecal PK Analysis

**Figure 1.**
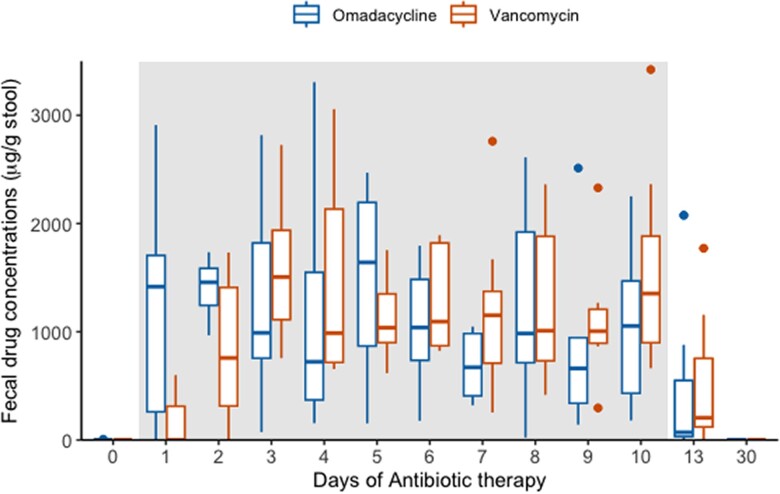
Fecal drug concentrations of omadacycline and vancomycin.

All stool samples were categorized as type 4 or lower on the Bristol stool chart. Of the 7 stool samples provided by the omadacycline group on day 1, 85.7% (6/7) demonstrated omadacycline concentrations >10 µg/g stool, and 100% (8/8) concentrations >900 µg/g stool by day 2 (Figure 1). By contrast, 57.1% (4/7) of day 1 stool samples from the vancomycin group had no measurable vancomycin concentrations; 28.6% (2/7) still had unmeasurable concentrations on day 2. By day 3 of antibiotic therapy, both groups had similar drug concentrations. Maximum mean concentrations were achieved on day 2 for omadacycline (4785 µg/g stool) and on day 9 for vancomycin (3990 µg/g stool). Daily averages of both antibiotics are shown in Supplementary Table 1.

### Gut Microbiota Analysis

**Figure 2.**
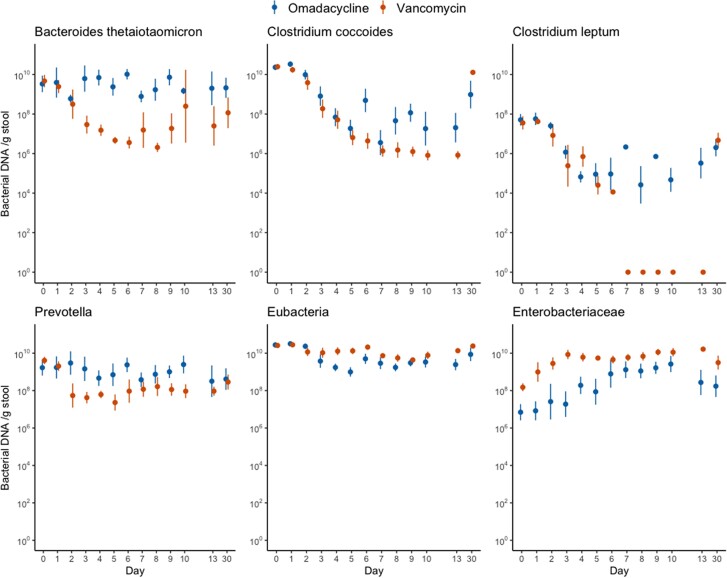
Quantitative changes of bacterial taxonomic groups measured by quantitative polymerase chain reaction. The y-axis of 10^0^ indicates below detection limit.

**Figure 3.**
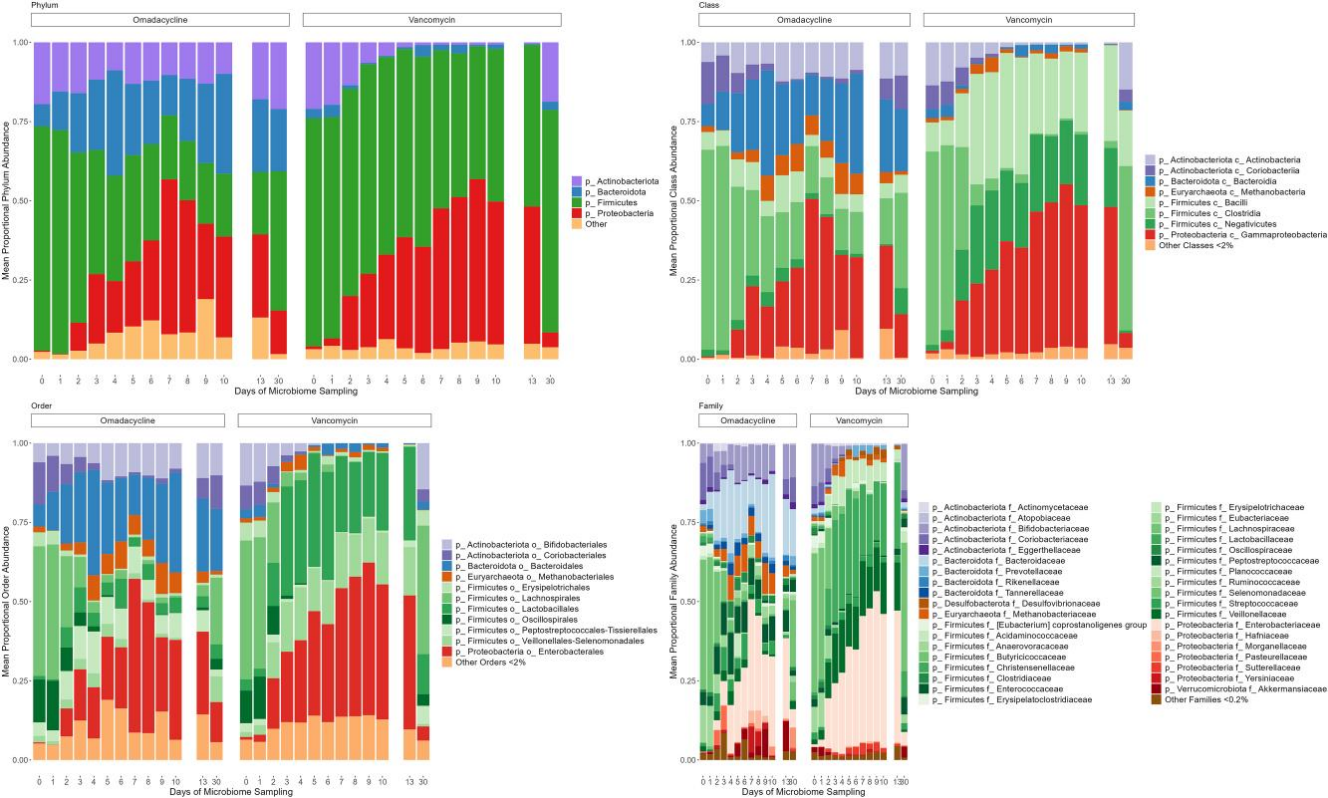
Relative abundance changes of omadacycline (left on each panel) and vancomycin (right on each panel) by phylum, class, order, and family; “o_” and “p_” refer to names of order (o) and phyla (p).

**Figure 4.**
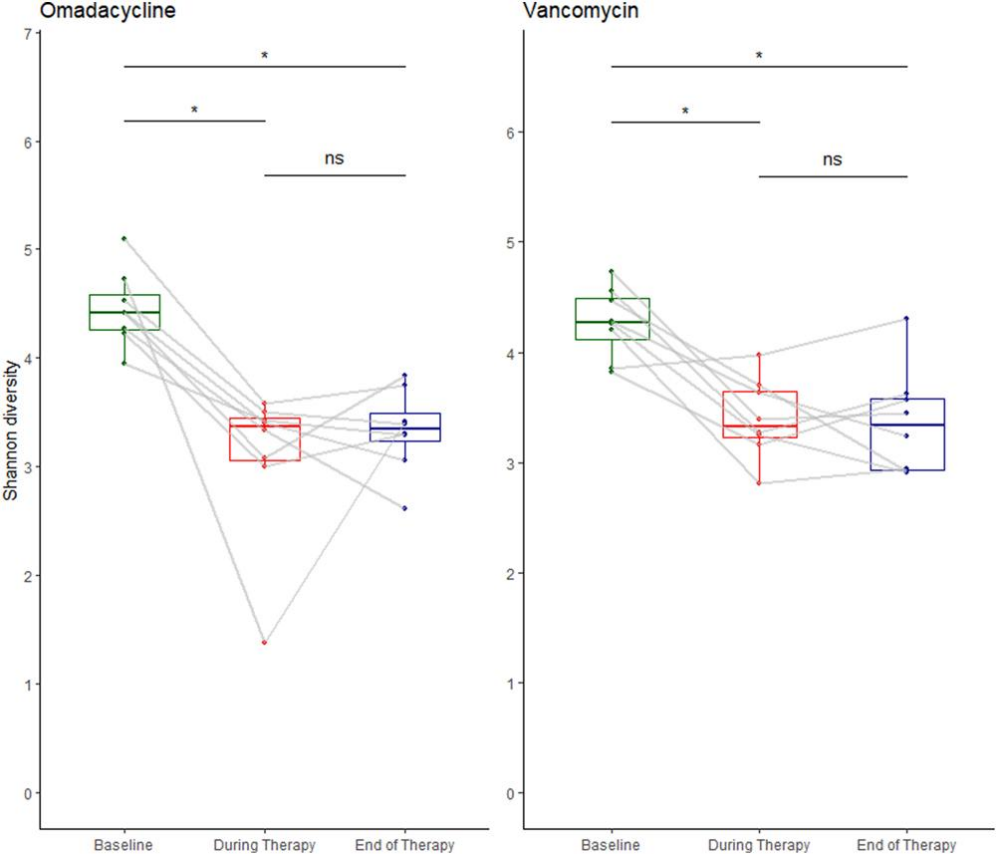
Alpha diversity changes of omadacycline and vancomycin as measured by Shannon diversity index between 3 different time points: baseline, during therapy, and end of therapy. *p < 0.05. Abbreviation: ns, not significant.

**Figure 5.**
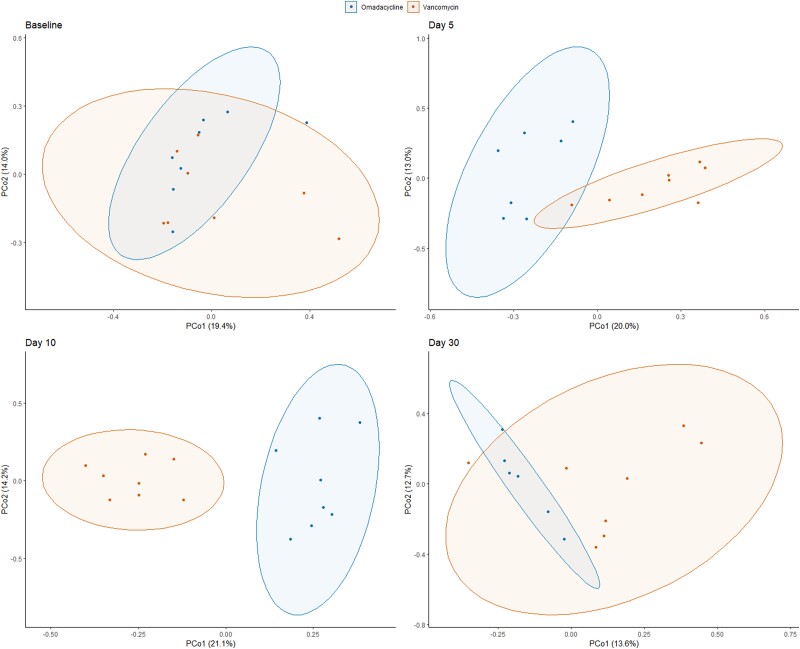
Beta diversity as measured by Bray–Curtis dissimilarity at baseline, day 5, day 10, and day 30. Abbreviations: PCo1, principal coordinate 1; PCo2, principal coordinate 2.

A total of 272 fecal samples from the 16 subjects were included for qPCR and DNA metagenomic analyses. Taxonomic groups measured by qPCR were similar on day 0, but marked decreases in *Bacteroides thetaiotaomicron*, *Clostridium coccoides*, *Clostridium leptum*, and Prevotella spp were observed in both omadacycline and vancomycin groups, although less pronounced in the omadacycline group (Figure 2). Enterobacteriaceae increased similarly in both antibiotic groups. The overall metagenomic analysis is shown in Figure 3. A significant proportional decrease in the Firmicutes phylum was observed in the omadacycline (−5.5 ± 0.6%; *P* < .0001) and vancomycin (−2.9 ± 0.4%; *P* < .0001) groups. In the omadacycline group, this was most commonly due to decreases in the Lachnospiraceae (−4.1 ± 0.4%; *P* < .0001) and Ruminococcaceae (−1.2 ± 0.2%; *P* < .0001) families. In the vancomycin group, similar proportional decreases were observed in Lachnospiraceae (−4.1 ± 0.4%; *P* < .0001), Peptostreptococcaceae (−0.2 ± 0.05%; *P* < .0001), and Ruminococcaceae (−1.0 ± 0.2%; *P* < .0001) with increases in Lactobacillaceae (+1.7 ± 0.5%; *P* < .05) and Veillonellaceae (+1.1 ± 0.3%; *P* < .0001) families. Actinobacteria phylum was also significantly reduced in the omadacycline (−0.7 ± 0.3%; *P* < .05) and vancomycin (−2.1 ± 0.2%; *P* < .0001) groups, most commonly due to a decreased proportion of Coriobacteriaceae in both groups (−1.1 ± 0.1%, *P* < .0001 in omadacycline; −0.6 ± 0.1%, *P* < .0001 in vancomycin) and a distinct decrease of Bifidobacteriaceae (−1.3 ± 0.2%; *P* < .0001) in the vancomycin group. A significant proportional increase in Proteobacteria phylum abundance was observed in the omadacycline (+3.7 ± 0.7%; *P* < .0001) and vancomycin (+5.0 ± 0.4%; *P* < .0001) groups due to an increase in Enterobacteriaceae family in both groups (*P* < .05). Alpha diversity was compared between 3 different times points: baseline, during therapy, and end of therapy (Figure 4). Compared to baseline, Shannon diversity decreased significantly during therapy in both the omadacycline (*P* = .0078) and vancomycin (*P* = .016) groups and continued to be significantly reduced at the end of therapy compared to baseline in those receiving omadacycline (*P* = .0078) and vancomycin (*P* = .025). Daily Shannon and Simpson diversity changes are shown in Supplementary Figures 1 and 2, with considerable intersubject variations observed in both groups (Supplementary Figures 3 and 4). Beta diversity showed that these shifts in microbial communities were significantly different between groups (Figure 5). The principal coordinate analysis revealed that the baseline samples between omadacycline and vancomycin groups were highly similar (ellipses representing 95% confidence bounds for each cluster). However, different antibiotic receipts resulted in distinct ellipses (on day 5 and 10) exhibiting significant microbial diversity differences between groups. By day 30 (follow-up visit), overlapping ellipses between antibiotic groups were observed.

## DISCUSSION

CDI is one of the most common healthcare-associated infections in the US, with limited available antibiotic treatment choices. Oral vancomycin, the most frequently used guideline-recommended agent in clinical practice, is associated with high recurrence rates and reports of reduced susceptibility against *C difficile* [21–25]. Thus, a clinically urgent need to develop new antimicrobial agents with *C difficile* activity and distinct mechanisms of action still remains. Omadacycline, an aminomethylcycline tetracycline, is an attractive option to repurpose as a CDI-directed antibiotic as it has potent in vitro activity against *C difficile*, a novel mechanism of action compared to current CDI-directed antibiotics, and a low propensity to cause CDI as shown in clinical trials. In this study, we demonstrated that oral omadacycline rapidly achieves high fecal concentrations. Using DNA sequencing metagenomics and qPCR quantification, we also showed that omadacycline produces a distinct microbial ecosystem compared to vancomycin, including the preservation of key Firmicutes groups. Both agents were well tolerated with no serious AEs. However, there were 4 episodes of vomiting in the omadacycline group.

Oral omadacycline has a bioavailability of 34.5%, does not undergo significant hepatic metabolism, and is primarily eliminated unchanged as an active drug in the feces [26]. Omadacycline achieved high and rapid fecal concentrations compared to the vancomycin group in which half had no measurable concentrations in 24 hours and 25% continued to have unmeasurable levels by day 2. Despite conflicting with previous results, we demonstrated similar findings in a prior study [27]. This delayed detection of fecal vancomycin may be due in part to its poor systemic absorption (bioavailability ∼0%) and its need to thus traverse the entire intestinal tract prior to entering the colon. Two previous studies have also demonstrated low vancomycin fecal concentrations in some patients with CDI given 125 mg 4 times daily that was not observed at higher doses. Whether a loading dose of vancomycin should be considered in patients with CDI will require further study [28, 29]. In contrast, a single dose of omadacycline 300 mg achieved rapid, measurable fecal concentrations in 24 hours of administration in 5 of 6 healthy subjects, although wide intersubject variability was noted [30]. Another study using a rat model and ^14^C-omadacycline revealed that the modes of fecal excretion largely involved biliary excretion (40%) and direct gastrointestinal secretion (30%) [31]. High concentrations in the colonic wall were observed within 5 minutes of IV infusion and maximum concentrations were observed at 7 hours after oral ingestion. Taken together, these multiple mechanisms of entry to the colon likely explain the rapid and measurable concentrations of omadacycline compared to vancomycin and provide a PK advantage as a CDI-directed antibiotic. Future studies with IV omadacycline will help confirm these results and provide PK evidence for IV omadacycline as a CDI-directed antibiotic. Both vancomycin and omadacycline persisted at high stool concentrations for several days after discontinuation of antibiotics, a finding relevant for dosing of fecal microbiota transplants and loss of *C difficile* colonization resistance [32].

Omadacycline has a broad spectrum of activity against anaerobic and aerobic bacteria [33]. Not surprisingly, both vancomycin and omadacycline resulted in significant changes in species richness and diversity, with proportionally reduced Firmicutes, Bacteroidetes, and Actinobacteria phyla in both groups compared to baseline. Within Phyla, distinct differences were noted in the proportional types of bacterial effected between omadacycline- and vancomycin-treated subjects. Two distinct findings from our analyses may help explain the clinical finding that omadacycline has a low propensity to cause CDI. First, beta diversity analyses demonstrated distinct microbial diversity changes between subjects given vancomycin and omadacycline. Second, in qPCR analysis, we demonstrated that certain commensal groups were less affected by omadacycline than vancomycin. This dysbiosis associated with vancomycin causes changes to primary bile acid and microbiota-accessible carbohydrate metabolism, which increases the risk of CDI [34, 35]. Whether the specific taxa changes seen following omadacycline receipt cause less disruption to these metabolic functions will need further study, but may explain why omadacycline and tetracyclines in general have a low risk of CDI development.

Our study has several limitations. We used vancomycin as a comparator CDI antibiotic, so future studies including fidaxomicin will be needed. This was a healthy volunteer study of relatively young subjects without diarrhea or gut permeability issues. Omadacycline use in patients with CDI will be needed to confirm and extend our results. The observed high and rapid increase in stool concentrations of omadacycline may be due to the loading dose used in this study. Whether the same concentrations would be observed without the loading dose will require further study. We used 16S rRNA sequencing for metagenomic analysis and so were unable to assess for antimicrobial resistance genes or species variants. As above, functional metabolomics such as short-chain fatty acid or bile acid studies will be needed to assess the biologic significance of the observed metagenomic changes.

## CONCLUSIONS

Oral omadacycline given to healthy subjects resulted in rapidly detectable fecal concentrations and gut microbiota diversity changes that are distinct from those seen following vancomycin receipt. These findings may help explain its low risk to cause CDI and warrants further development as a CDI-directed antibiotic.

## Supplementary Data

Supplementary materials are available at *The Journal of Infectious Diseases* online (http://jid.oxfordjournals.org/). Supplementary materials consist of data provided by the author that are published to benefit the reader. The posted materials are not copyedited. The contents of all supplementary data are the sole responsibility of the authors. Questions or messages regarding errors should be addressed to the author.

## Supplementary Material

jiad537_Supplementary_Data

## Notes

***Acknowledgments.*** K. W. G. contributed to study design, acquisition and analysis of data, and interpretation of results and reviewed and revised the manuscript. J. J. contributed to the acquisition and analysis of data and interpretation of results and drafted, reviewed, and revised the manuscript. C. H., K. B., W. W., T. M. L., S. A., B. M. H., H. A., C. L., M. J. A., and A. G.-L. contributed to the acquisition and analysis of data and interpretation of results. All authors approved the final version of the submitted manuscript.

***Financial support.*** This work was supported by Paratek Pharmaceuticals, Inc, as an investigator-initiated grant (grant number G0505124). Funding to pay the Open Access publication charges for this article was provided by Paratek Pharmaceuticals.
