# Supplementary material for: Fecal Pharmacokinetics and Gut Microbiome Effects of Oral Omadacycline Versus Vancomycin in Healthy Volunteers
Source: J Infect Dis. 2023 Dec 5;229(1):273–81. doi: 10.1093/infdis/jiad537 (PMC10786255; doi:10.1093/infdis/jiad537)
Supplement: jiad537_Supplementary_Data [file jiad537_supplementary_data.zip › HVM omdvan MS_v5_JID_clean.docx]

Supplemental Figure 1. Alpha diversity changes over the study period as measured by Shannon diversity index. Omadacycline is shown in blue and vancomycin in orange.

Supplemental Figure 2. Alpha diversity changes over the study period as measured by Simpson diversity index. Omadacycline is shown in blue and vancomycin in orange.

Supplemental Figure 3. Subject-specific alpha diversity changes as measured by Shannon diversity index. Blue dots reflect subjects given omadacycline and orange dots reflect those given vancomycin. HVM = Healthy Volunteer Microbiome study

Supplemental Figure 4. Subject-specific bacterial abundance changes over the study period HVM = Healthy Volunteer Microbiome study
