# Supplementary material for: Fecal Pharmacokinetics and Gut Microbiome Effects of Oral Omadacycline Versus Vancomycin in Healthy Volunteers
Source: J Infect Dis. 2023 Dec 5;229(1):273–81. doi: 10.1093/infdis/jiad537 (PMC10786255; doi:10.1093/infdis/jiad537)
Supplement: jiad537_Supplementary_Data [file jiad537_supplementary_data.zip › Supplemental Table.docx]

Supplemental Table 1. Fecal concentrations of omadacycline and vancomycin by antibiotic administration day

| **Day of antibiotic therapy** | **Mean concentration (µg/g stool), range** | |
| --- | --- | --- |
|  | **Omadacycline** | **Vancomycin** |
| 0 | 0 ± 0 (0-0) | 0 ± 0 (0-0) |
| 1 | 1,180 ± 1,064 (3-2,909) | 174 ± 257 (0-598) |
| 2 | 1,826 ± 1,148 (968-4,364) | 848 ± 696 (0-1,732) |
| 3 | 1,727 ± 1,517 (75-4,785) | 1,593 ± 713 (757-2,724) |
| 4 | 1,146 ± 1,116 (157-3,305) | 1,461 ± 953 (655-3,056) |
| 5 | 1,753 ± 1,109 (155-3,636) | 1,127 ± 380 (618-1,753) |
| 6 | 1,060 ± 530(178-1,794) | 1,313 ± 505 (823-1,892) |
| 7 | 687 ± 325 (322-1,047) | 1,205 ± 765 (255-2,760) |
| 8 | 1,270 ± 910 (24-2,611) | 1,283 ± 759 (418-2,361) |
| 9 | 875 ± 868 (143-2,512) | 1,537 ± 1,243 (296-3,990) |
| 10 | 1,080 ± 739 (182-2,251) | 1,578 ± 940 (822-3,422) |
| 13-14 | 474 ± 771 (3-2,076) | 522 ± 631 (0-1,771) |
| 30-32 | 0 ± 0 (0-0) | 0 ± 0 (0-0) |

Lower limits of quantification for omadacycline and vancomycin were 0.1 ng/mL and 0.4 µg/mL, respectively
