# Supplementary figures and images for: Fecal Pharmacokinetics and Gut Microbiome Effects of Oral Omadacycline Versus Vancomycin in Healthy Volunteers
Source: J Infect Dis. 2023 Dec 5;229(1):273–81. doi: 10.1093/infdis/jiad537 (PMC10786255; doi:10.1093/infdis/jiad537)

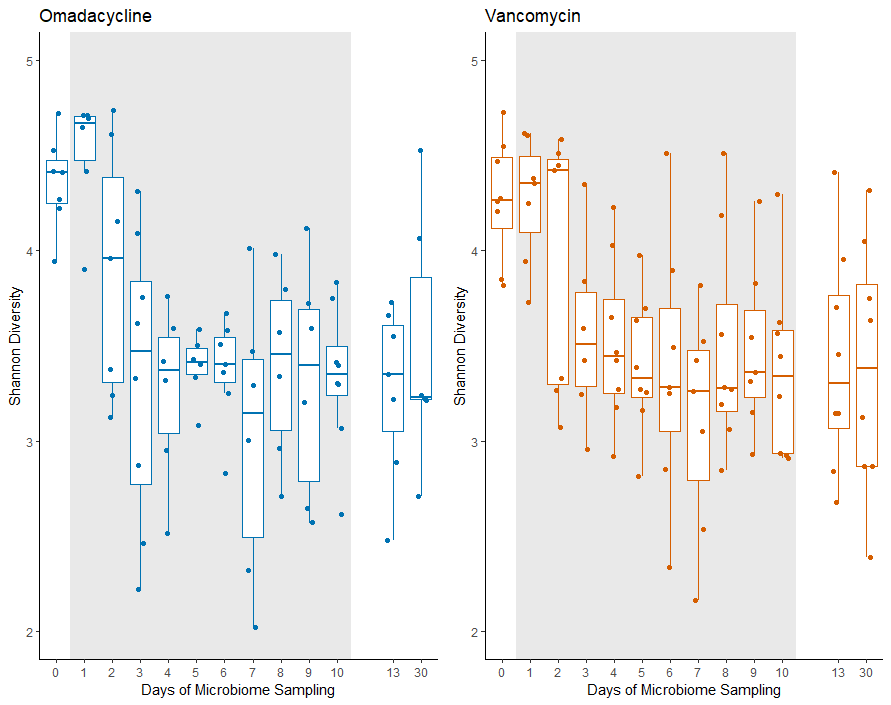

Supplement: jiad537_Supplementary_Data [file jiad537_supplementary_data.zip › Supp Fig 1. HVM_omdvan_Shannon_cb_final.tiff]

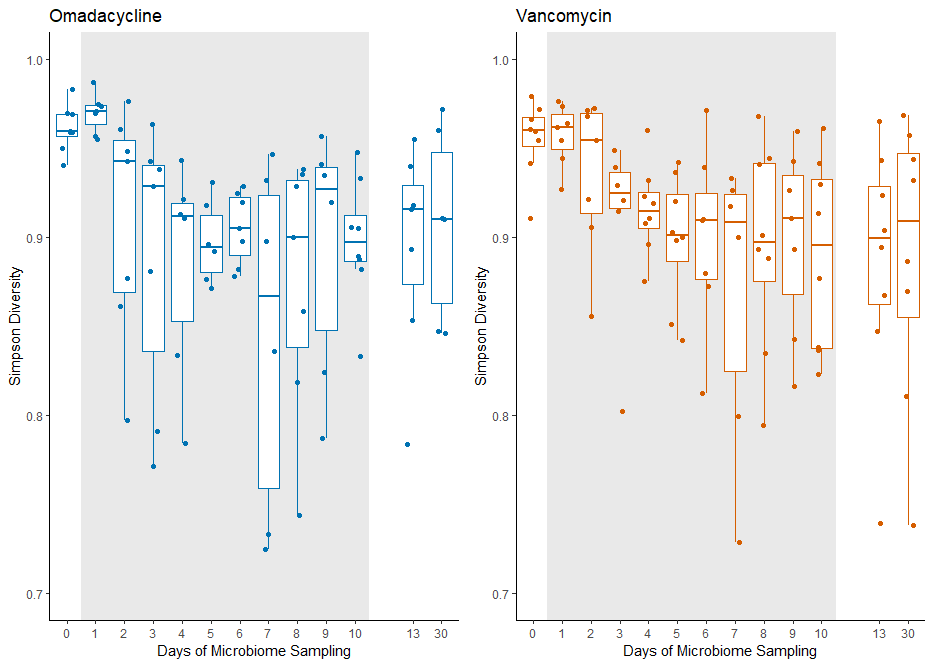

Supplement: jiad537_Supplementary_Data [file jiad537_supplementary_data.zip › Supp Fig 2. HVM_omdvan_Simpson_cb_final.tiff]

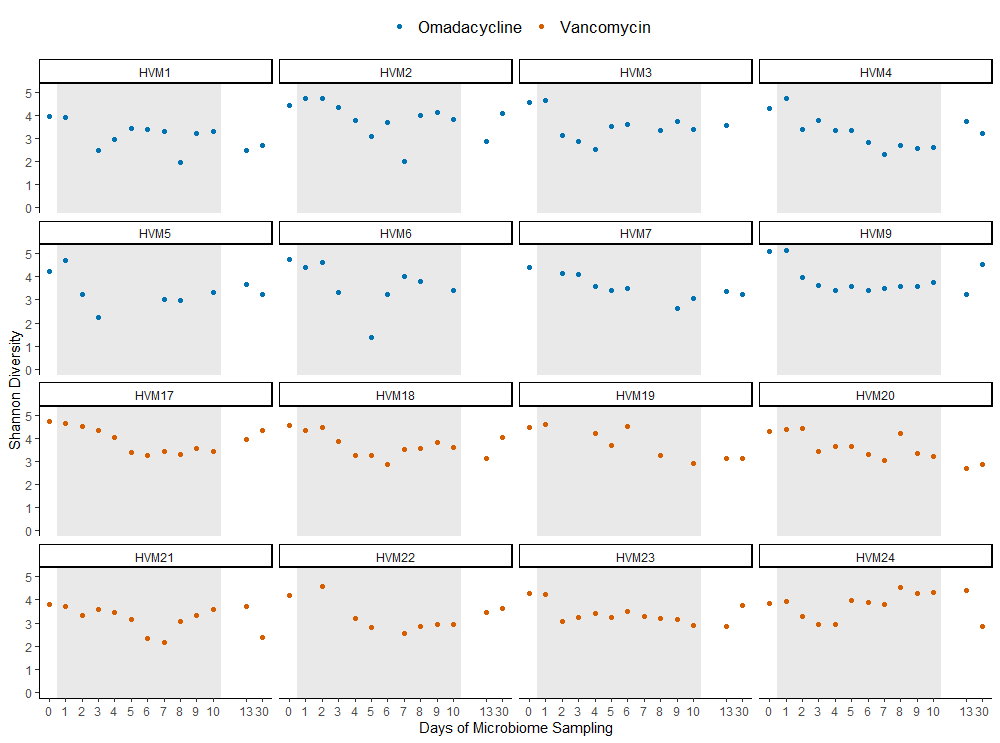

Supplement: jiad537_Supplementary_Data [file jiad537_supplementary_data.zip › Supp Fig 3. HVM_Subjects_Shannon_final.tiff]

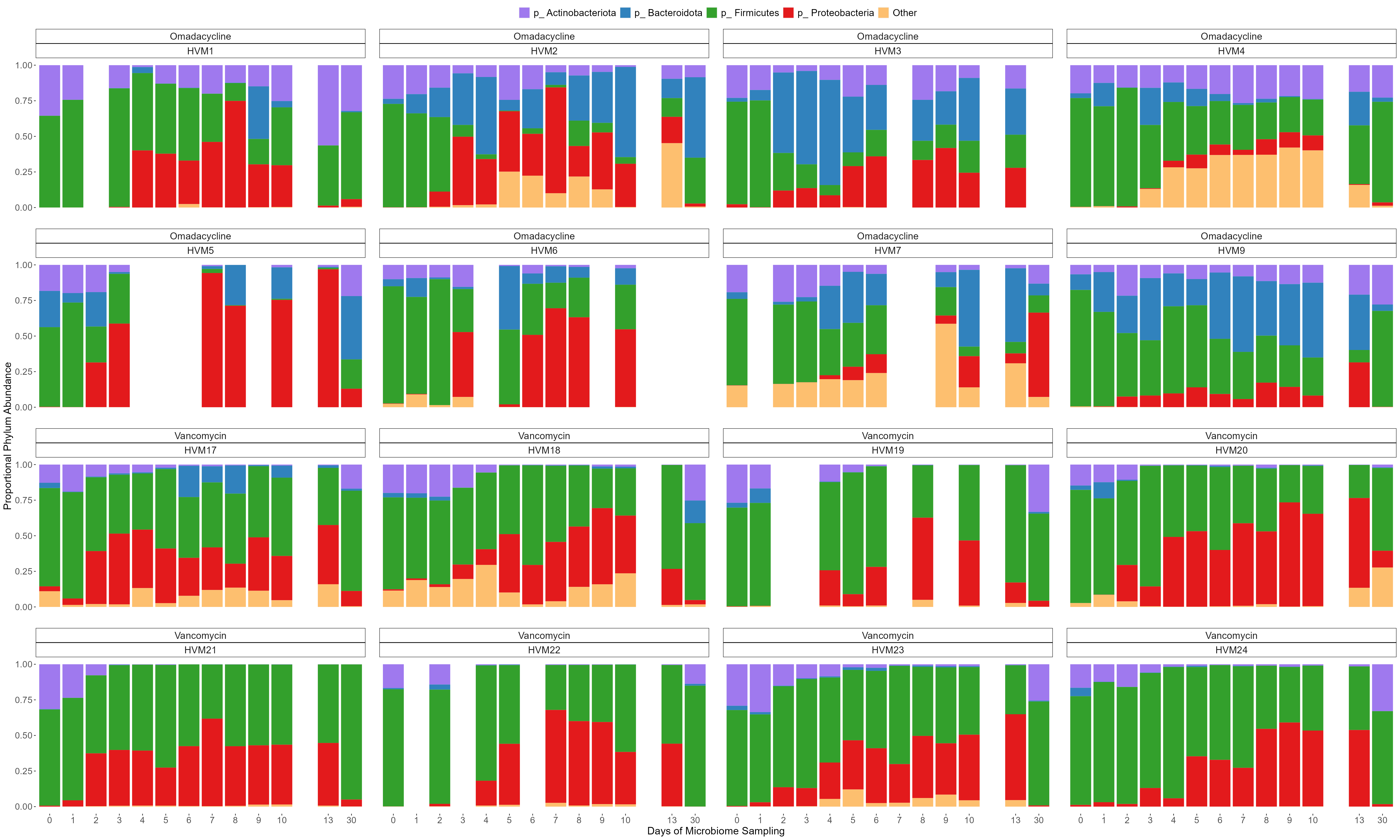

Supplement: jiad537_Supplementary_Data [file jiad537_supplementary_data.zip › Supp Fig 4. HVM_Subjects_Phylum_final_dpi200.tiff]
